# A Combined Method for Segmentation and Registration for an Advanced and Progressive Evaluation of Thermal Images

**DOI:** 10.3390/s141121950

**Published:** 2014-11-19

**Authors:** Emilio Z. Barcelos, Walmir M. Caminhas, Eraldo Ribeiro, Eduardo M. Pimenta, Reinaldo M. Palhares

**Affiliations:** 1 Department of Electrical Engineering, Federal University of Triangulo Mineiro, Av. Dr. Randolfo Borges Jr. 1250, Univerdecidade, CEP 38064.200, Uberaba, MG, Brazil; 2 Department of Electronics Engineering, Graduate Program in Electrical Engineering, Federal University of Minas Gerais, Av. Antonio Carlos 6627, Pampulha, CEP 31270.901, Belo Horizonte, MG, Brazil; E-Mails: caminhas@cpdee.ufmg.br (W.M.C.); palhares@cpdee.ufmg.br (R.M.P); 3 Department of Computer Sciences, Florida Institute of Technology, 150 West University Blvd, Melbourne, FL 32901, USA; E-Mail: eribeiro@cs.fit.edu; 4 Medical and Physiology Department, Cruzeiro Esporte Clube, R. Adolfo Lippi Fonseca, 251, Ceu Azul, CEP 31545.260, Belo Horizonte, MG, Brazil; E-Mail: empimenta@uol.com.br

**Keywords:** thermography, image segmentation, image registration, sports medicine

## Abstract

In this paper, a method that combines image analysis techniques, such as segmentation and registration, is proposed for an advanced and progressive evaluation of thermograms. The method is applied for the prevention of muscle injury in high-performance athletes, in collaboration with a Brazilian professional soccer club. The goal is to produce information on spatio-temporal variations of thermograms favoring the investigation of the athletes' conditions along the competition. The proposed method improves on current practice by providing a means for automatically detecting adaptive body-shaped regions of interest, instead of the manual selection of simple shapes. Specifically, our approach combines the optimization features in Otsu's method with a correction factor and post-processing techniques, enhancing thermal-image segmentation when compared to other methods. Additional contributions resulting from the combination of the segmentation and registration steps of our approach are the progressive analyses of thermograms in a unique spatial coordinate system and the accurate extraction of measurements and isotherms.

## 1. Introduction

Naturally invisible to the human eye, radiation in the infrared spectrum can be depicted by thermal cameras. These devices are precise temperature-measurement tools with current models featuring fine thermal sensitivities and diverse temperature ranges. With the increased availability of infrared thermal cameras and sensors, the analysis of thermal images (*i.e.*, thermograms) has found various applications in engineering [1–4], where inspecting equipment heat output prevents major faults or inefficiency, and in medicine [5–9], where the detection of irregular patterns in skin temperature may be linked to pathological conditions. The physiological relation between temperature and tissue health also underpins the application of thermal imaging in sports medicine, as musculoskeletal injuries are known to produce local changes in skin temperature [10,11]. These changes refer to local blood-flow variations that directly affect the surroundings' temperature and, indirectly, the skin's temperature by conduction. These variations can be hypo- or hyper-thermal (*i.e.*, reduced or increased temperatures).

**Figure 1.**
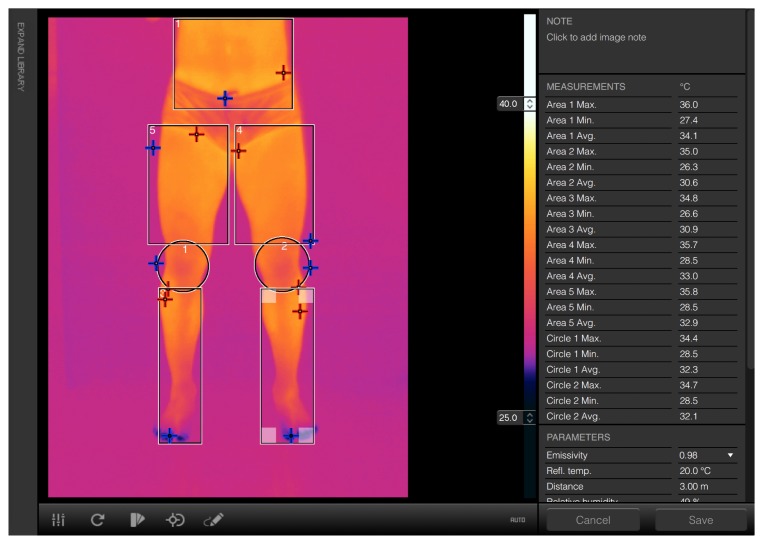
Screen shot of currently available thermal-analysis software. The selection of regions of interest (ROIs) is limited to simple rectangular and elliptical shapes. No functions are available for automatically segmenting the main subject from the background or processing sequences of images taken in distinct moments.

In this paper, the application of thermal-image analysis techniques favors the assessment, monitoring, and early detection of potential muscle injuries in high-performance athletes. Current practice in sports medicine uses thermal imaging for supporting the detection of musculoskeletal injuries by measuring temperature variations in specific body parts [12,13]. Specifically, the temperatures of selected body regions are measured using software provided by thermal-camera manufacturers. Using the software, healthcare professionals manually select simple-shaped regions (*i.e.*, rectangles and ellipses) within which temperature measurements are made. Common measurements include minimum and maximum temperatures, as well as region statistics, such as averages and standard deviations. Figure 1 shows the graphical user interface of a typical software for thermography analysis.

The use of thermograms for assessing muscular injury has a major advantage for being non-invasive and radiation free. The technique is not new, but it has seen renewed attention [14]. Examples of recent work include Hildebrandt *et al.* [15], who used thermography-based measurements for assessing potential knee injury in skiers. Their work compared the symmetrical pattern of temperature between the right and left knees as an indicator of fatigue or possible injury. Another recent and related work was done by Cuevas [16] on the use of thermography for assessing muscular fatigue in soccer players.

While thermography technology shows great potential as an assessment tool for preventing muscular injury, there are issues that need further attention. For instance, when analyzing body parts (e.g., quadriceps muscle) as a whole, rectangular and elliptical regions of interest may include some background information that can decrease the measurements' quality. Trying to avoid this issue means selecting smaller regions that might not completely cover the actual area of interest. The accurate assignment of specific regions may require manually selecting several smaller regions of interest, followed by the recalculation of averages and extrema. In fact, such a practice is not only time consuming, but also error prone. In addition, the analysis of how the heat patterns move and change in size over time is hard to perform, since images acquired in different moments will invariably present geometrical distortions due to misaligned spatial coordinate systems.

To address these problems, a combined method using image segmentation, processing, and registration techniques is proposed for an improved selection of regions of interest, resulting in more accurate measurements. Furthermore, the method provides the means for analyzing changes in thermal patterns over time by transforming images into the same spatial coordinate system. The proposed method is used as part of an injury-prevention program in collaboration with a major professional soccer club in Brazil.

Teams participating in the Brazilian main soccer league undergo an intensive playing and training schedule. During the tournament season, a team might play about 70 matches. Under such a demanding schedule and because of the limitation on the number of players allowed per tournament, injuries due to muscle fatigue are common. As a result, minimizing the occurrence of players' injuries from muscle fatigue is key to ensuring a club's successful campaign.

To help maximize athletes' physical readiness during the tournament season, clubs' medical personnel maintain continuous monitoring of the players' physical conditions. Monitoring procedures use a combination of periodical examinations of physiological indicators (e.g., blood samples), as well as general musculoskeletal-injury assessment.

Recently, the medical staff of the Brazilian soccer club Cruzeiro (*i.e.*, Cruzeiro Esporte Clube, in Portuguese) has been using thermography analysis to assist with the monitoring and diagnosis of muscle fatigue in players. Cruzeiro's procedure consists of imaging players after each soccer match or major practice and analyzing the images for changes in skin temperature surrounding target muscles.

Altogether, this paper's main contribution is to provide the foundations and technical means for: the automatic detection of adaptive body-shaped regions of interest, the accurate extraction of measurements and regions of homogeneous skin temperature (*i.e.*, isotherms), and the progressive analysis of thermograms corrected to a unique spatial coordinate system. The proposed method combines image analysis techniques, such as segmentation and registration, with the supervised selection of parameters to produce precise information on spatio-temporal variations in sequences of thermograms and respective isotherms, favoring a more advanced investigation of the athletes' conditions along the competition.

## 2. Method

**Figure 2.**
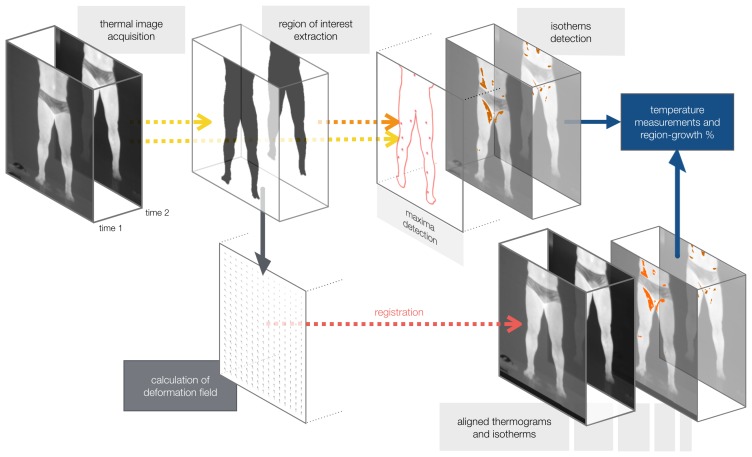
Main steps of the proposed method.

The proposed approach has five specific steps: (1) the thermal-imaging acquisition protocol; (2) the segmentation of the athletes' bodies; (3) the extraction of isotherms; (4) the conversion from sensed radiant energy to temperatures; and (5) the registration of players' thermograms (and associated isotherms) of distinct moments for temporal analysis. Figure 2 presents the proposed method's workflow. The diagnosis should be held by a sports medicine professional, and it is not part of our method.

### 2.1. Thermal Imaging Acquisition Protocol

The dataset consists of 348 thermal images of 28 volunteering players from Cruzeiro. The thermal images of each player's lower thorax and limbs were acquired in an anteroposterior manner (*i.e.*, frontal and dorsal views). Volunteers signed an informed-consent form, and the protocol was approved by the ethics committee of the Federal University of Minas Gerais under registration number ETIC–291/09. Furthermore, the acquisition follows the regulations established by the Brazilian National Health Council and standard thermography-related protocols.

The first stage of the acquisition process was to establish a standard for each player. Here, images were taken after 33 days of the start of the soccer season. Then, during the season, thermal images were collected after the end of each match, but only from athletes who played at least 2/3 of games. For all acquisitions, players were advised not to perform physical activity for 40 h before the thermograms were captured. The medical team also registered the maximum, minimum, and average temperature values from quadriceps, ischiotibials, gastrocnemius, and tibialis, on the left and right sides.

Images were acquired in an acclimatized room of 20 °C and with a relative humidity of 65%. Players stayed in the room for ten minutes prior to the exam for thermal equilibrium. Then, athletes stood at a 2.5 m distance from the camera, and anterior and posterior thermal images were taken. Materials consisted of a FLIR T420 thermal camera connected to a computer with the ThermaCam Researcher Pro software and a digital thermo-hygrometer (to monitor room temperature and humidity). The camera was periodically calibrated by accredited third-party thermography professionals for quality and reliability purposes.

**Figure 3.**
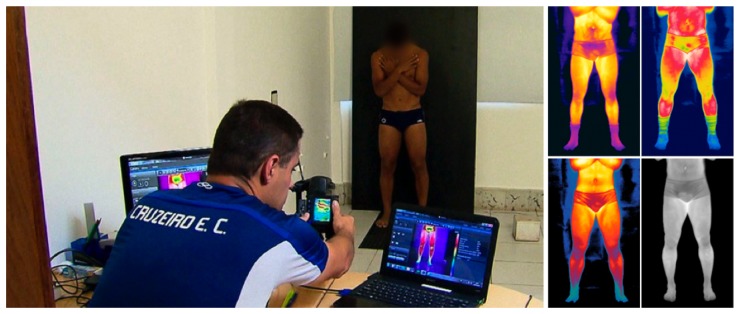
**(Left)** The thermal imaging acquisition protocol; **(Right)** Examples of players' thermograms, using different color maps.

Despite the established protocol, features that differ between acquisitions were observed. For example, some players wore socks during the image acquisition. The protocol setup and example images using different color maps are shown in Figure 3. Actual thermal images are grayscale, similar to the one shown on the bottom-right corner of Figure 3, but with much lower contrast.

### 2.2. Image Segmentation (ROI Extraction)

Segmentation aims to separate an area, namely the region of interest, or simply ROI, from the remainder of the image content, or background [17,18]. Here, the ROI is the athlete's body.

Instead of using simple and manually selected geometric shapes as regions of interest, the proposed method automatically extracts masks using the actual body shape of the players (*i.e.*, silhouettes).

To extract the initial body mask, we modified the segmentation method proposed by Otsu [19]. Given an image containing pixels belonging to two main classes: foreground and background regions, Otsu's method calculates the probability distribution of pixel intensities and then finds the optimal threshold *t_otsu_* that separates the classes by maximizing the between-class variance.

**Figure 4.**
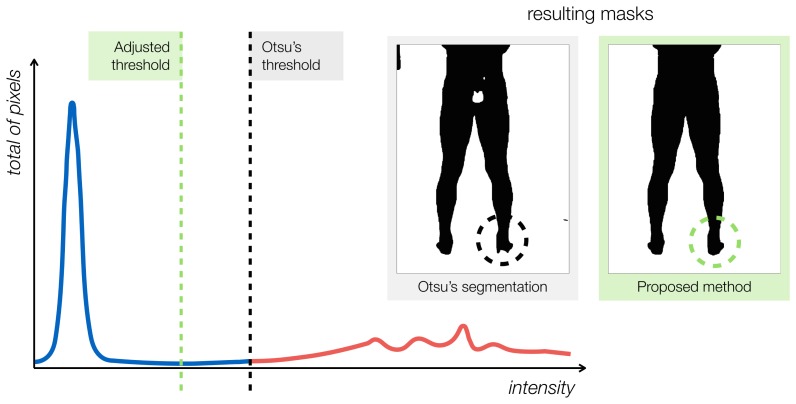
Finding the mask silhouette of the athlete's body, automatically. An adjusted threshold is used so that more pixels are included in the ROI (body-mask). The black dashed line is Otsu's optimal threshold. The green dashed line is our method's adjusted threshold. Picture insets illustrate the effect of adjusting the threshold (*i.e.*, the foreground region is expanded, correcting segmentation near the feet areas) and removing small artifacts.

Otsu's method works well for separating the athletes' bodies from the room's background. However, due to the nature of the thermal information and the physical phenomena of conduction and convection, the object of interest and background will likely share a subset or range of the overall distribution, where only the radiometric value is not enough for its classification. Any threshold-based segmentation method, such as Otsu's, will not handle this issue properly. Furthermore, the resulting segmentation may contain artifacts, such as holes and smaller spurious regions. To address these problems, a correction factor is applied to the original Otsu's threshold, so that more pixels are included in the foreground region. The new threshold is t* = *φ* t_otsu_, where *φ* is the correction factor. Figure 4 illustrates the concept.

In the figure, the threshold calculated by the original Otsu's algorithm is represented by the black dashed line. The blue and red graph-contours represent the background and foreground regions, respectively. The segmented mask produced using Otsu's threshold has missing parts, as indicated in the foot region (left-hand side inset in Figure 4). The corrected threshold fixes most of the the missing parts. The proposed ROI-extraction method concludes by using connected-component analysis [20] to close holes and remove spurious regions of small areas.

### 2.3. Isotherms Detection

In the collected thermograms, skin regions that display abnormal temperature patterns may indicate muscle injury. These regions form clusters of pixels of similar temperatures, called isotherms. Either hot or cold isotherms are preferred depending on the type of condition analyzed [5].

A thermal image is a quantized radiometric map of sensed energy, *R*(x), and can be seen as a surface that contains peaks (*i.e.*, high-temperature) and troughs (*i.e.*, low-temperature). The proposed approach automatically extracts isotherms by first detecting local extrema in the radiometric map *R*(x). These detected locations are the input to a multi-seeded region-growing scheme. In this section, the extraction of hot isotherms is described. The procedure for extracting cold isotherms is analogous, but instead of detecting peaks, it finds troughs.

Here, local extrema are detected by comparing each pixel value inside the ROI to the average of the values within a squared-neighborhood of a side of *n* pixels. It is essential to have a squared mask with an odd pixel size, so that it can be balanced, and the pixel under analysis is indeed the mask's center of gravity. Furthermore, the value of the centered pixel should not be considered in the average calculation. In this scenario, the pixel under analysis is considered a peak if its intensity value is higher than the average of its neighbors in the squared mask. A trough is found in the opposite manner. For each neighborhood, multiple detections are pruned, and only the most prominent peak is kept. As *n* grows, less extrema are expected to be detected. Furthermore, observe that the set of extrema found by a mask of a higher area is a subset of an equivalent squared mask with a smaller side in pixels. The maxima detection can be accomplished by means of the grayscale morphological operation of dilation with a flat structuring element [21].

**Figure 5.**
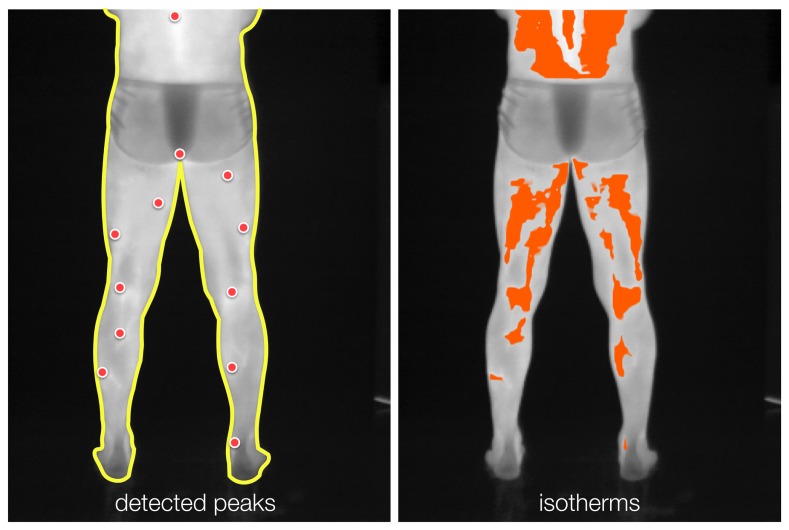
Extracting isotherms. **(Left)** Automatically detected peaks (red dots) serve as seed points; **(right)** hot isotherms are extracted using region growing controlled by a user-defined threshold, for flexibility.

The detected peaks serve as starting points for growing radiometric cluster regions [22]. These regions are incrementally expanded in a breadth-first manner by including locations for which radiometric values are below a given threshold (*i.e.*, a percentage of the associated peak's energy). At the end of the region-extraction process, small-area and spotty regions are removed. Figure 5 shows the detected peaks of radiometric values and the corresponding (hot) isotherm resulting from the proposed method.

### 2.4. Converting the Raw Thermal Data to Temperatures

Once the isotherms have been extracted, the proposed method calculates temperature measurements. While the temperature is proportional to radiometric measurements, the relationship between the two quantities is nonlinear. The spectral radiant exitance of a perfect blackbody as a function of its temperature and the wavelength of the emitted radiation [23] is given by Planck's law:

(1) $$M_{\text{BB}}\left(\lambda ,T\right)=\frac{2\pi hc^{2}}{\lambda^{5}}{\left[\exp\left(\frac{hc}{\lambda kT}\right)-1\right]}^{-1}\times 10^{-4}W/\left(m^{2}\mu \text{m}\right)$$

where, *λ* is the wavelength, *T* is the temperature, *h* is the Planck constant, *c* is the speed of light, and *k* is the Boltzmann constant.

Then, the total radiant exitance from a blackbody at a given temperature can be found by integrating the spectral exitance (Equation (1)) over all wavelengths, which yields:

(2) $$M_{\text{BB}}\left(T\right)=\int_{0}^{\infty }M_{\text{BB}}\left(\lambda ,T\right)d\lambda =\sigma T^{4},$$

where *σ* = 2*π*^5^*k*^4^/15*c*^2^*h*^3^ ≈ 5.67 × 10^−8^ W/(m^2^K^4^) is the Stefan-Boltzmann constant. Numerous materials are considered to have the properties of graybodies, even though real surfaces are not truly graybodies over the whole electromagnetic spectrum [24]. For these objects in general, the total radiant exitance is defined by:

(3) $$M\left(T\right)=\varepsilon M_{\text{BB}}\left(T\right),$$

where *ε* ∈ (0, 1] is a material-specific emissivity coefficient (e.g., for human skin [14], *ε* = 0.98).

The thermal camera used stores radiometric data as a two-dimensional matrix *R* of 320 × 240 pixels of thermal resolution. At each pixel location x = (*x*, *y*)^(x022A4)^, the value *R*(x) is the radiant energy of objects sensed by the camera, quantized in 16 bits (*i.e.*, 65,536 intensity values). The exact values of the quantized radiometric data depend on the camera's calibration parameters and settings. This information is stored in the image-file metadata. Given the metadata parameters and the *R*(x) values, the temperature can be determined as a function of the emissivity and the radiometric values by inverting Equation (3).

### 2.5. Deformable Image Registration for Thermal Images

**Figure 6.**
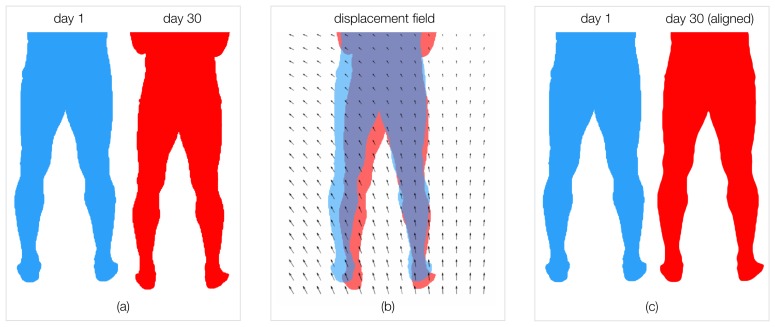
Registration of thermograms of a player, acquired one-month apart. (**a**) Masks at Days 1 and 30; (**b**) unregistered masks superimposed and the displacement field; (**c**) mask at Day 30 aligned with the coordinate system of the mask at Day 1.

Because of the interest in measuring how isotherm regions change over time, the proposed method superimposes the athlete's thermograms onto a single coordinate frame by using deformable image registration. This step removes geometric distortions in the acquisition process. Here, a standard nonrigid registration method based on optical flow [25,26] is used. The players' segmented body silhouettes (*i.e.*, binary masks) are used as the input for the registration, as presented in Figure 6.

Given two silhouette masks *S*_1_(x) and *S*_2_(x) of the same player taken at different times, the method estimates the best two-dimensional displacement field that transforms the first mask into the coordinate system of the second, *i.e.*:

(4) $$\text{u}\left(\text{x}\right)=\arg\underset{\text{u}}{\min}\left\{\left\|(S_{1}\left(\text{x}\right)-S_{2}\left(\text{x}+\text{u})\right)\right\|+\alpha {\left\|\nabla \text{u}\right\|}^{2}\right\},$$

where $S_{2}^{\prime }\left(\text{x}\right)=S_{2}\left(\text{x}+\text{u}\right)$ is the displaced version of *S*_2_(x). The term ‖∇u‖^2^ enforces smoothness in the resulting displacement field and also helps reduce folding effects [26]. The minimization problem in Equation (4) is solved using the Levenberg-Maquardt optimization procedure, which is well suited for solving these types of functions. Using other optimization methods, such as conjugate gradient descent and interior-point algorithm is expected to produce similar results. Then, given the displacement field obtained from Equation (4), one can transform the detected isotherms (from Section 2.3) and also the acquired thermograms (*i.e.*, radiometric maps). The steps are summarized in Algorithm 1.


**Algorithm 1** Registration of isotherms and thermograms acquired in distinct moments.
**Require:** Thermograms (*i.e.*, radiometric maps) *R*_1_ and *R*_2_, according to protocol (Section 2.1).
1: Extract the body silhouettes *S*_1_ and *S*_2_ using image segmentation (Section 2.2): {*S*_1_, *S*_2_} ← **ExtractBodyMasks**(*R*_1_, *R*_2_)2: Obtain isotherm maps *I*_1_ and *I*_2_ from radiometric data from pixels inside masks (Section 2.3): {*I*_1_, *I*_2_} ← **DetectIsotherms**((*R*_1_, *S*_1_), (*R*_2_, *S*_2_))3: Estimate the displacement field **u** that aligns silhouette *S*_2_ to silhouette *S*_1_ (Section 2.5): **u** ← arg min_u_ (‖ (*S*_1_(x) − *S*_2_(**x** + **u**)) ‖ + *α*‖ ∇u‖^2^)4: Align isotherms in *I*_2_ to isotherms in *I*_1_ and radiometric map *R*_2_ to radiometric map *R*_1_:
$I_{2}^{\prime }\leftarrow I_{2}\left(\text{x}+\text{u}\right)$ and 
$R_{2}^{\prime }\leftarrow R_{2}\left(\text{x}+\text{u}\right)$


## 3. Experiments

Experiments were conducted in order to evaluate the proposed method on sequences of thermograms taken from professional soccer players from Cruzeiro. This section is subdivided into specific sets of trials that partially portray the most relevant steps of the proposed approach. In the first part, the goal is to present the method's ability in fine segmenting the athletes' body silhouettes from the background in an automated manner, even with the presence of general artifacts as cited earlier in the text. Then, the following experiments indicate how using image segmentation followed by non-rigid registration allows for more accurate progressive analyses when compared to standard measurements made within unregistered and simple-shaped regions of interest.

Recall from the previous section that the players' thermal images were taken after each soccer match along the season. Images consisted of anterior and posterior views of the lower abdomen and legs. In total, the method was applied to 348 thermal images obtained from 28 volunteering athletes. Data collection followed the acquisition protocol described in Section 2.1.

The method extracted the body silhouette from each player's thermogram. This step was performed using the segmentation procedure described in Section 2.2. Here, the correction factor *φ* = 0.985 was used. It was determined after experimentation on sample images, as discussed later in this section.

The following step was to detect the isotherms inside the body-silhouette regions (Section 2.3). First, a set of salient points (e.g., local maxima and minima) were spotted inside the body area, using the mask as the region of interest. The detected local extrema were then used as seeds for the region-growing algorithm to produce the isotherms. The criterion used to indicate the end of the region-growth process was a range of 0.2% of the radiant energy of the peak under analysis.

In their original coordinate frames, the thermograms and detected isotherms contain shape deformations originating from viewpoint changes and other environmental distortions in the image-acquisition process. Here, the person-specific body masks were used as input to the nonrigid registration method (Section 2.5) to obtain displacement fields, **u**(**x**). These displacement fields were then used to register all thermal images and extracted isotherms to the coordinate frame of the first-acquired image (standard) of each player, for a suitable spatio-temporal analysis.

### 3.1. On the Automatic Selection of Regions of Interest

Related applications with thermal imagery have mainly reported the manual selection of regions of interest, and no technique has yet been presented for the automatic extraction of ROIs as proposed in this work. Nonetheless, our method is tested against two other techniques for comparison purposes.

**Figure 7.**
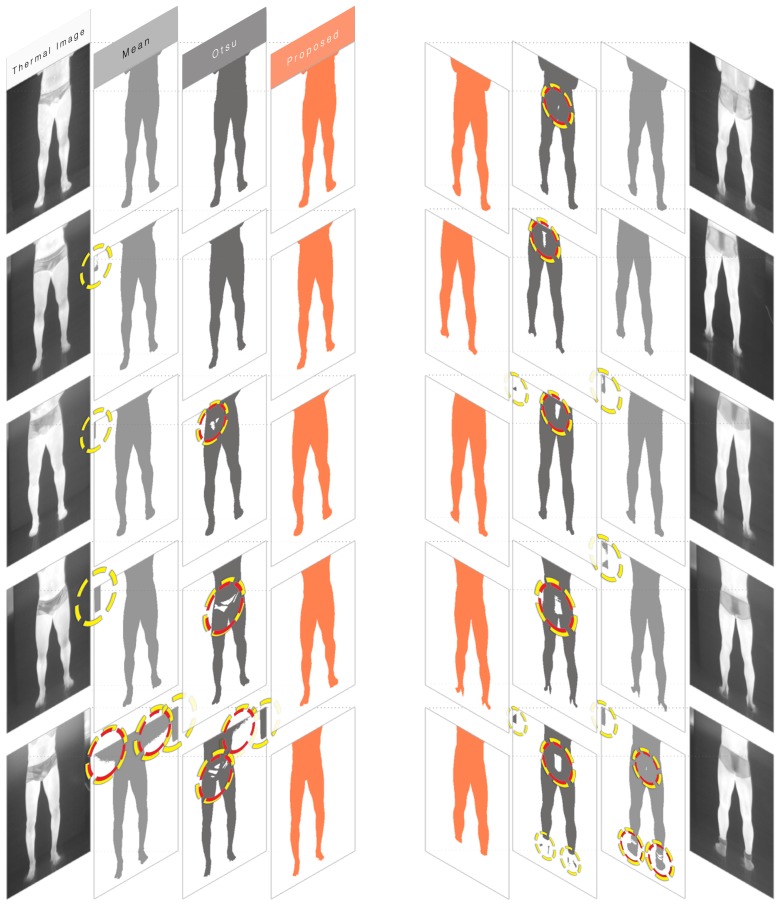
Experiment on the automatic selection of regions of interest. Ten thermograms from different players were selected at random. Three masks were automatically extracted using the mean method (in light gray), Otsu's method (in dark gray), and the segmentation method proposed by the authors (highlighted in orange). Issues in the masks are indicated in dashed yellow circles. Problems with the main ROI are highlighted with dashed red circles. The proposed method consistently presents a precise, well-delimited, and less noisy outcome.

Ten thermograms were arbitrarily selected from the dataset for this trial. Among the elected thermograms, exactly five frontal and five dorsal images were chosen. Furthermore, all images were from different players. For each thermogram, three masks were extracted. The first mask was the result from segmenting all pixels whose radiometric value exceeded the image's overall mean. The second mask was the result from Otsu's method. Finally, the third mask is the outcome of the proposed approach, as presented in Section 2.2. Figure 7 shows the results, where dashed circles indicate artifacts in the segmentation processes (*i.e.*, holes, over-segmentation). Artifacts that overlapped with the main region of interest are highlighted in red. The artifact occurrence percentages are shown in Table 1.

### 3.2. On the Progressive Analysis of Thermograms

Instead of setting strict requirements regarding the athletes' positioning in the acquisition protocol or pre-established regions of interest, the proposed method accurately aligns thermograms into the same spatial coordinate system for a more advanced progressive analysis of the players' conditions.

**Figure 8.**
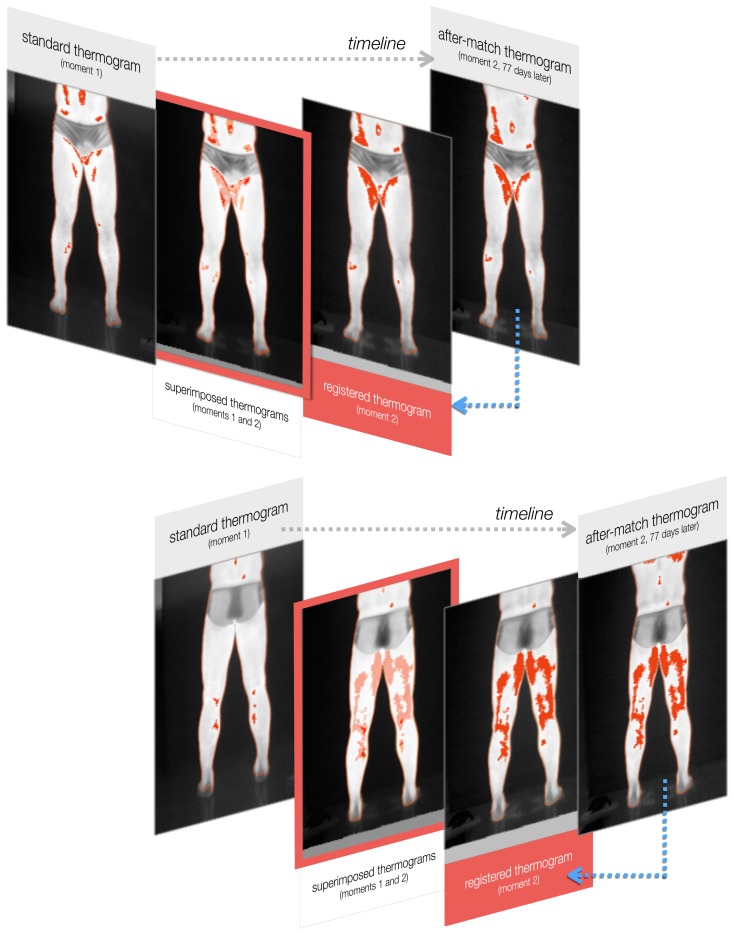
Experiment illustrating the registration of two frontal and two dorsal thermograms of the same athlete, captured 77 days apart. For each set of tests, the left and right images are the original thermograms. The third image is the result of the proposed registration method. The second image (in a red frame) is an overlay of the original thermogram in moment *m*_1_ with the registered thermogram of moment *m*_2_. This approach allows for the assessment of the variations among thermograms and the extraction of the region-growth percentage.

Figure 8 shows an example of the outcome produced after the registration, as detailed in Section 2.5. Anterior and posterior images of a volunteering athlete are presented. Thermal images were acquired in arbitrary moments *m*_1_ and *m*_2_, 77 days apart. Four images are portrayed in each test. On the left and right ends, the original thermal images taken at moments *m*_1_ and *m*_2_ are respectively presented. They are both superimposed by their masked-isotherms, in red. The ROIs in moments *m*_1_ and *m*_2_ serve as the input for the registration method. The transformation that takes them to the same coordinate system is calculated iteratively. The third picture of each sequence is the resulting radiometric image and isotherm from transforming moment *m*_2_. Note that this approach retains the original shape of one image in the coordinate system of the other. The second picture in the sequence is a superimposition of the first and third thermograms to highlight the variations and to evaluate the region-growth percentage.

**Figure 9.**
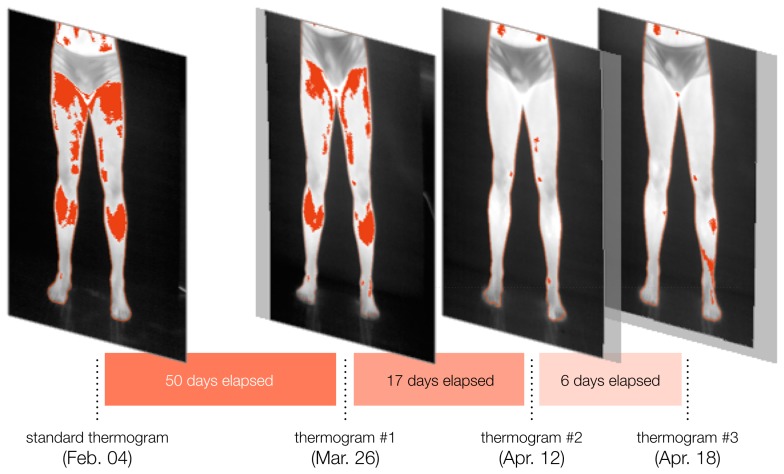
Example experiment on the progressive analysis of a sequence of thermograms of a volunteering player during the beginning of the season. The evolution of the player's fitness is assessed qualitatively, through the observation of the registered images, and quantitatively, based on the region-growth percentage.

Finally, a sequence of thermograms of the same athlete is presented in Figure 9. The figure illustrates the progressive analysis of the player's condition throughout part of the tournament.

## 4. Discussions

The results from the segmentation experiments in Section 3.1 indicate the quality of the proposed method when compared with two other automatic segmentation methods.

The mean-threshold method is the simplest of them and the least computationally expensive. However, it is very context-dependent and can present unstable behavior, as seen in the fifth frontal test in Figure 7. On the other hand, Otsu's method can be computationally costly, since it performs an extensive search while finding its optimal threshold. For 16-bit depth images, such as the thermograms used in the tests, most algorithms make a simplification conversion to an eight-bit depth, which consequently degrades the results' quality. This was not the case in these experiments, as all values in the 16-bit range were considered by our implementation of Otsu's method. Still, this presented the worst quantitative results for the test images.

The proposed method outperformed the other techniques. It managed to be robust to environmental changes, shifts in the thermal camera's position, and the presence of undesired artifacts. Indeed, the robust, yet flexible, quality of the proposed segmentation approach is based on the statistical features in Otsu's method combined with a correction factor and post-processing techniques that are suitable for thermal imagery. All of the masks resulting from our method presented better quality than their counterparts from the other methods, producing a precise, well-delimited, and less noisy outcome.

Given the acclimatized nature of the room and considerably consistent acquisition protocol, a single correction could be established for the image dataset. The value of the correction factor *φ* is adjustable and can be changed by the medical analyst if needed.

**Figure 10.**
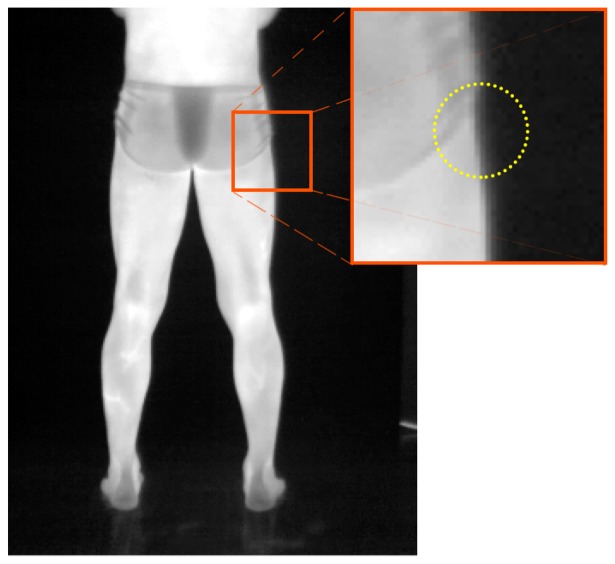
The fading effect of the intensity levels of the radiometric image near the edges of the human subject, due to the surface curvature. This is an expected concern in thermal imaging, and its counter-effects can be disregarded in this application.

Regarding the curvature of the human body and its effects on the temperature measurements and ROI extraction, Lahiri [5] has indicated that surface curvature is not an issue in medical applications, except for female breast imaging. Therefore, although the emissivity coefficient is dependent on the angle of the emitting radiation to the surface's normal, considering it to be constant is adequate here. Clearly, the edges of the human body will present angles that are almost orthogonal to the camera. Figure 10 illustrates the effect, which yields different intensity values and fading temperatures near the edges. This common behavior in infrared thermography does not seem to affect the proposed method, since the segmentation step presented optimal results when extracting the regions of interest.

**Figure 11.**
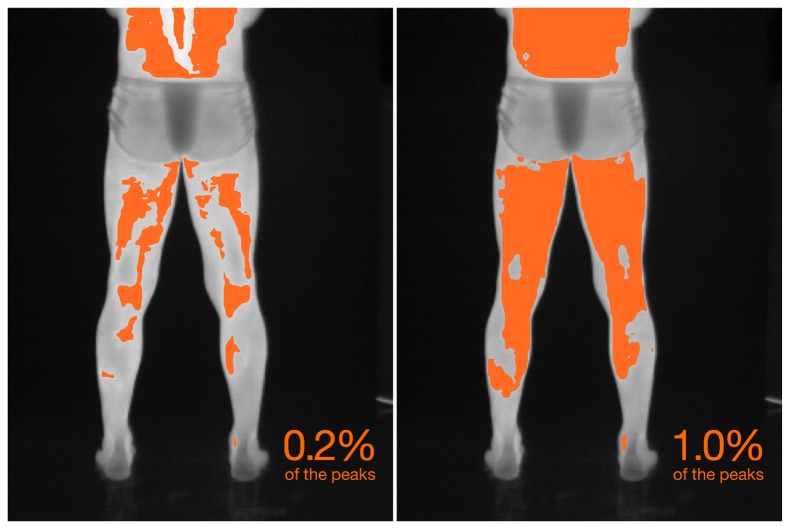
The effect of the region growing stoppage criterion on isotherm extraction. While the ability to adjust this parameter can be useful for medical analysis and provide some flexibility, setting it inadvertently may lead to undesirable results.

The setting of some parameter values based on experimentation is a limitation of the proposed method. These parameters include the segmentation correction factor and the region-growth stoppage criterion. The latter is very application dependent. Considering the thermograms presented in this article, the 16-bit sampling allows for 65,536 different levels of sensed energy. Isotherms are defined as regions where temperature values occur within a closely-related range. Thus, considering the camera's temperature range specifications, that the subject under investigation is a human body, and that the background is in equilibrium with the environment, most sensed values will fall in a fraction of the range of possible intensities. For selected thermograms, the radiometric values generally fell into an effective range of 4%–12% of the full radiometric range. A stoppage value of 0.2% of the peak corresponds approximately to a subrange of 1%–2.5% of the effective range. Therefore, should the criterion grow, the isotherms may cover the full subject body, considering the human thermoregulatory system. Figure 11 presents the effect of the stoppage constraint on isotherm extraction.

While the ability to adjust these parameters in the proposed approach allows for fine tuning by medical analysts, we plan to address the automatic selection of these values in future work by using computer intelligence techniques.

The registered isotherm maps show how skin temperature measurements for corresponding body regions vary over time. Looking at the registered isotherms shown in Figures 8 and 9, it can be noted that some spotted areas were matched between acquisition times, although changes are evident. Measuring changes in region-growth percentage and mean temperature variation is important for the analysis of thermography over time, and the proposed method makes this possible through image registration.

In the example results shown in Figure 8, specific hot patterns close to the player's umbilicus and inner thighs were found in isotherms in both acquisition times. Furthermore, hot regions near the gastrocnemius and adductor magnus muscles were detected in the dorsal test. Here, the analysis can be both qualitative, by assessing the sequence of registered thermograms, and quantitative, by evaluating temperature measurements, isotherms, and the region-growth percentage, for example. An overall increase in the isotherm area might indicate the player's fitness evolution or injury-prone conditions, yet the final conclusions and diagnoses should always be performed by sports medicine professionals.

In contrast with previous works on applying thermogram analysis to injury assessment, the proposed method is able to extract patterns of temperature variations that occur over periods of time. While our method does not actually produce a diagnosis, its results were used by Cruzeiro's medical team for assisting with the detection of potential injury and with the monitoring of the treatments' progress.

Altogether, this approach was robust to image artifacts caused by differences in room temperature and issues such as players wearing socks or sandals. Partial occlusion of the torso and small variations caused by player's motion or camera repositioning did not seem to affect the method's outcome.

## 5. Conclusions

A method for the progressive analyses of sequences of infrared thermograms was presented with an application for monitoring and preventing injury-related thermal patterns in high-performance athletes.

The method combines image segmentation, processing, and registration techniques to produce qualitative and quantitative information on spatio-temporal variations of thermal images and their isotherms. The segmentation and registration steps are key to improving the accuracy of measurements extracted from the thermal images. Without such an approach, analyzing the changes of temperature patterns over time might be inadequate. By restricting measurements to be taken only from inside the body's silhouette, the method ensures that temperature measurements are not corrupted by values lying outside the expected region of interest.

Experiments indicated the method's ability in robustly extracting precise regions of interest from the radiometric data, even when artifacts and undesirable objects were present. Furthermore, the original use of image registration for the progressive analyses of thermograms was found to be very effective, allowing for a more advanced and precise investigation, and relevant, as it can be extended to foster other thermography-based applications.

The presented approach was tested on thermograms of soccer players as part of an injury-monitoring program developed in collaboration with the Brazilian professional soccer team, and national champions, Cruzeiro. The team's medical staff used the data produced by our method to assist with the prevention and monitoring of their players' muscular injuries. Recall that any diagnosis should be held by a sports medicine professional and is not part of the proposed approach.

Future directions include the automatic labeling of body parts and anatomic regions in thermograms by using the registration method in [27] in combination with an anatomical atlas and the investigation of machine-learning techniques for analyzing spatio-temporal patterns that appear in isotherms in the context of sports injuries. Research is underway and will be reported in due course.

## Figures and Tables

**Table 1.** Comparing different methods for automatically extracting regions of interest from ten arbitrarily selected thermograms. The resulting numbers are from experiments in Figure 7.

**Method**	**(% of Trials with) Suitable ROI**	**(% of Trials with) Issues Outside ROI**	**(% of Trials with) Issues Inside ROI**
Mean	30%	70%	20%
Otsu	20%	30%	80%
Proposed method	100%	0%	0%
